# Private Practitioners’ Perspectives on Their Involvement With the Tuberculosis Control Programme in a Southern Indian State

**DOI:** 10.15171/ijhpm.2016.52

**Published:** 2016-05-08

**Authors:** Solomon Salve, Kabir Sheikh, John DH Porter

**Affiliations:** ^1^Department of Global Health and Development, London School of Hygiene and Tropical Medicine, London, UK.; ^2^The Maharashtra Association of Anthropological Sciences, Centre for Health Research and Development (MAAS-CHRD), Savitribai Phule Pune University, Pune, India.; ^3^Public Health Foundation of India, New Delhi, India.; ^4^Departments of Clinical Research and Global Health and Development, London School of Hygiene and Tropical Medicine, London, UK.

**Keywords:** Public Sector, Private Sector, Private Practitioners (PPs), Public-Private Mix (PPM), Tuberculosis (TB), India

## Abstract

**Background:** Public and private health sectors both play a crucial role in the health systems of low- and middle-income countries (LMICs). The tuberculosis (TB) control strategy in India encourages the public sector to actively partner with private practitioners (PPs) to improve the quality of front line service delivery. However, ensuring effective and sustainable involvement of PPs constitutes a major challenge. This paper reports the findings from an empirical study focusing on the perspectives and experiences of PPs towards their involvement in TB control programme in India.

**Methods:** The study was carried out between November 2010 and December 2011 in a district of a Southern Indian State and utilised qualitative methodologies, combining observations and in-depth interviews with 21 PPs from different medical systems. The collected data was coded and analysed using thematic analysis.

**Results:** PPs perceived themselves to be crucial healthcare providers, with different roles within the public-private mix (PPM) TB policy. Despite this, PPs felt neglected and undervalued in the actual process of implementation of the PPM-TB policy. The entire process was considered to be government driven and their professional skills and knowledge of different medical systems remained unrecognised at the policy level, and weakened their relationship and bond with the policy and with the programme. PPs had contrasting perceptions about the different components of the TB programme that demonstrated the public sector’s dominance in the overall implementation of the DOTS strategy. Although PPs felt responsible for their TB patients, they found it difficult to perceive themselves as ‘partners with the TB programme.’

**Conclusion:** Public-private partnerships (PPPs) are increasingly utilized as a public health strategy to strengthen health systems. These policies will fail if the concerns of the PPs are neglected. To ensure their long-term involvement in the programme the abilities of PPs and the important perspectives from other Indian medical systems need to be recognised and supported.

## Background

Public and private health sectors both play a crucial role in the health systems of low- and middle-income countries (LMICs).^1-3^ The terms public sector and private sector can be defined in several different ways.^4^ In general, “the ‘public sector’ includes organizations or institutions that are funded by state revenue and that function under government budgets.”^5^ In contrast, the ‘private sector’ comprises those organizations and individuals that are privately owned and operate outside of government authority.^6^ Over the last two decades, researchers focusing on health systems and health policy issues have emphasised the untapped potential of the private medical sector in LMICs.^7-11^ In 2010, the 63rd World Health Assembly passed a resolution to strengthen the capacity of governments to constructively engage the private sector to provide essential healthcare services.^12^ Efforts to involve private players reflect the larger movements in public health towards strengthening health systems.

In India, the health system is broadly divided across the public and the private sectors. As enshrined in the Indian constitution, healthcare delivery is largely the responsibility of the provincial states and territories, rather than the federal government. Running in parallel with the public health sector, India has one of the most highly privatized healthcare systems in the world, and this system fills the gaps in the public health system. According to the National Family Health Survey-3, the private medical sector remains the primary source of healthcare for 70% of households in urban areas and 63% of households in rural areas.^13^ The private healthcare sector is highly diverse, ranging from: practitioners of Western medicine (MBBS doctors); those trained in Indian systems of medicine often termed as ‘AYUSH’ (an acronym for Ayurveda, Yoga, Unani, Siddha, and Homeopathy); and those without formal trainings. According to the Central Bureau of Health Intelligence, in 2012 there were 883812 qualified allopathic doctors, and 628634 AYUSH doctors registered.^14^ Public health researchers writing on universal health coverage (UHC), have emphasised ‘quality improvement and reduction of out-of-pocket expenditure on healthcare through a well-regulated integration of the private sector within the national healthcare system.’^15^ It is clear that both sectors have their strengths and weaknesses and that neither can replace the other, nor can they alone achieve the best results for the health system.^5^ The Ministry of Health and Family Welfare (MoHFW) has adopted the strategy of public-private partnership (PPP) as an attempt to strengthen the health systems, by developing national policies and guidelines to emphasise the engagement of all healthcare providers in different National Health Programmes.

The vision of the Government of India for tuberculosis (TB) control is a “TB-free India” with a reduction in the burden of the disease until it is no longer a major public health problem.^16^ To achieve this vision, the Revised National TB Control Programme (RNTCP) has adopted a new objective to achieve ‘universal access’ to quality diagnosis and treatment for all TB patients in the community.^17^ One of the key pillars towards achieving universal access is to ‘expand efforts to engage all care providers.’^16^ The World Health Organization’s (WHO’s) ‘post-2015 TB strategy’ strongly emphasises private sector involvement for achieving universal coverage.^18^

Irrespective of these international and national attempts around partnerships, achieving sustained partnership between the public and private health sectors, through sustaining interest, motivation and involvement of private practitioners (PPs) in disease programmes (both communicable and non-communicable), continues to be a major challenge for the concerned health authorities.^5,19,20^ This paper provides insights into how to sustain the involvement of PPs in disease control programmes by focusing on their perspectives and experiences around the implementation of the public-private mix (PPM) policy for TB control in India.

## Policy Context: Public-Private Mix-Tuberculosis

### Global and National Tuberculosis Burden

Worldwide, TB is the cause of millions of deaths annually, 83% of which occur in three WHO regions: South East Asia, the Western Pacific, and the African Region.^21^ The WHO Global Tuberculosis Report for the year 2013, estimated that there were 8.6 million new cases of TB (13% living with HIV) and 1.9 million deaths from the disease in 2012 alone.^21^ India is ranked 17th out of the 22 countries with the highest burden of TB.^22^ In 2011, of the estimated 9 million global annual incidence of TB, 2.3 million occurred in India^23^ – one quarter (26%) of the global burden of TB.^21^ From 1961 to 1993, India implemented an integrated national TB programme (NTP). In 1993, the Government of India revised the NTP to include the WHO DOTS policy, and the programme was re-named the ‘RNTCP.’^24,25^ Since its inception in 1996, the programme has initiated the treatment of more than 12.8 million patients, and has saved nearly 2.3 million lives.^26^

### Private Medical Sector and Tuberculosis Management

In 1999-2000, the WHO undertook an assessment of the role of private healthcare providers in TB control all over the globe. The assessment revealed that in low-income countries a large proportion of TB suspects and cases accessed private healthcare services but these practitioners were often not associated or connected to the public health system.^27^ In India, the private medical sector plays a significant role in the management of TB.^28^ Secondly, there are reports that, in addition to the public sector, a huge private sector in Andhra Pradesh manages substantially high number of patients for TB.^29^ A survey in 2007 showed that 80% of the PPs had seen TB patients in Hyderabad and most of them were managing TB on own.^30^ Studies of patient management practices have highlighted inappropriate prescribing, inadequate counselling, and violations of clinical and ethical guidelines on patient care,^31-34^ as well as reported patient delays in accessing DOTS.^35^ These management practices affect patients’ adherence to treatment (due to their limited ability to afford treatment) and increase the development of drug resistance resulting from incomplete and inadequate treatment.^36,37^ Despite the irrational and inequitable practices, PPs remain an important and the preferred providers of primary care for many people^38^ because of their accessibility in terms of distance and opening hours, their responsiveness to patients, their maintenance of privacy and confidentiality, and due to the general poor quality of the public sector services.^39,40^

### India’s Response to Public-Private Mix-Tuberculosis Policy

Linking TB control with the private sector has been an important policy development at WHO and in September 2000, WHO created the PPM DOTS – PPM for DOTS expansion strategy.^41^ WHO defined PPM for TB control as ‘strategies that link all healthcare entities within the private and public sectors (including health providers in other governmental ministries) to national TB programmes for expansion of DOTS activities.’^42^

In response to the WHO’s PPM strategy, the RNTCP piloted and documented innovative PPM DOTS models.^43^ From 2002, they expanded PPM DOTS activities nationwide using the policy guidelines for involvement of non-governmental organizations (NGOs)^44^ and PPs^45^ under different schemes.^46^ The Mahavir Project in Hyderabad, Andhra Pradesh, was the first initiative to formally involve private providers in the RNTCP.^47,48^ This led the RNTCP to further recognize the need to partner with NGOs and private healthcare providers^43^ to create an integrated health system to control TB. Several PPM DOTS initiatives have demonstrated that involving PPs and NGOs can to help increase TB case detection.^48-50^

However, over the past decade the uptake of PPM-TB schemes, under a formal government agreement, has declined. In addition, the emergence of multi-drug resistant TB (MDR-TB) and HIV-TB co-infection has posed a huge challenge for effective TB programme implementation.^16^ As a result, there was a need to revise the existing PPM-TB policy schemes to meet the challenges of present day programme implementation. Since 2008, these revised schemes have been disseminated in all the States.^51,52^

### Role of Private Practitioners in Public-Private Mix-Tuberculosis Policy Implementation

The PP can get involved in a single activity in the TB programme or in multiple activities depending on his or her capacity, interest and the requirements of the programme. To be a DOTS provider, PPs need to show willingness to place DOTS box for TB patients at his/her clinic. A box of medications for full course of treatment is reserved for every registered patient. The PPs are expected to ensure follow-up sputum collection and late patient retrieval, as well as to maintain RNTCP records for the patients and to permit on-site monitoring by RNTCP supervisory staff as per RNTCP guidelines. In addition to being a DOT provider, PPs can refer those individuals they suspect of having TB for diagnosis and treatment, irrespective of whether the client is diagnosed as having TB at a private labs. DOTS providers receives an honorarium of Rs 250 per case (US$3.9) for each TB case treatment that is successfully completed, and in the case of MDR-TB, the amount increases to Rs 1000 per case (US$15.9).^52^

### Contribution of Private Practitioners to Case Detection

In 2003, based on the results of the feasibility studies, the RNTCP launched an intensified PPM project in 14 urban areas in India. Additional human resources were provided to each of these sites in the form of an RNTCP medical consultant and two field workers.^53^ Medical consultants were reported to be useful in faster implementation of DOTS as compared to areas without consultants.^54^ In order to further mainstream the PPM-DOTS, additional human resources provided to these 14 sites had been withdrawn by January 2008,^55^ and attempts continue to be made to scale-up PPM-DOTS.^51^ Although, the scale-up of PPM implemented in the 14 cities is claimed to be productive,^56^ a critical review of the RNTCP annual reports over the last seven years^26,51,55,57-60^ shows that the contribution by PPs to the referral of TB suspects in these 14 sites has remained around only 4% (Figure).

**Figure:**
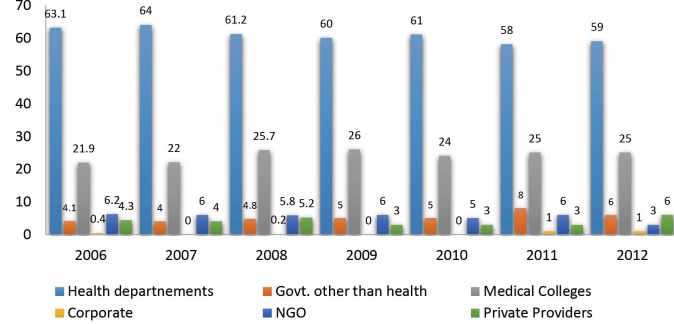
(TB) Suspects Referred to RNTCP Microscopy Centres. Abbreviations: NGO, non-governmental organization; TB, tuberculosis; RNTCP, Revised National TB Control Programme. The 14 sites are large urban areas in 14 different states: Bihar (Patna), Chandigarh, Gujarat (Ahmadabad), Jharkhand (Ranchi), Karnataka (Bangalore), Kerala (Thiruvananthapuram), Madhya Pradesh (Bhopal), Maharashtra (Pune-Mumbai), New Delhi, Orissa (Bhubaneswar), Rajasthan (Jaipur), Tamil Nadu (Chennai), Uttar Pradesh (Lucknow), and West Bengal (Kolkata).

Considering that 80% of India’s practicing doctors work in the private medical sector^39^ and that they alone manage 60% of the TB case load,^28^ the referrals from PPs under PPM-TB is minimal. One explanation may suggest that since patients’ first access PPs’ services, most patients in the DOTS enrolment would be de facto referrals from private providers,^47^ and perhaps the public health sector is failing to capture these informal referrals.^61^ Another perspective may involve a consideration of the extent that the widely advocated PPM strategy has been adapted to the needs of private providers and participating NGOs, and are these partnerships sustainable?^62^

Since 2007, the RNTCP has been working closely with the Indian Medical Association (IMA) to increase the number of PPs collaborating with the national programme.^55^ In addition, attempts to collaborate with AYUSH practitioners are being undertaken through the ‘Axshya’ India project.^63^ Despite these efforts, there is no substantial guidance on how to improve and sustain the PPM-TB policy through involvement of PPs. This paper attempts to address these identified gaps, by focusing on the individual practitioners; how they understand and think about the policy, how they feel about their involvement, and what challenges and constrains they identify within the policy guidelines.

## Methods

To understand the process of implementation of the PPM-TB policy the study used a cross-sectional study design, with qualitative data collection and analysis within an ethnographic approach.^64^ The findings emerge from in-depth interviews conducted with PPs, from participant observation in TB clinics, and from reflective process of field notes. Data collection was carried out between November 2010 and December 2011 in a district, in a Southern State of India. To safeguard the anonymity and confidentiality of the respondents the study site will be left unnamed.

The work was conducted in a TB unit (TU). Under the RNTCP, the study district has been divided into nine TUs each covering an approximate population of 0.5 million. Each TU consists of four to five designated microscopy centre’s (DMC’s) each covering a population of 0.1 million. In consultation with the District Programme Managers, one of the TUs was selected as an area for the detailed exploration of the PPM-TB policy implementation. The selection of PPs was restricted to the study TU. For this study, “PP” is defined as a physician who practices regularly in individual clinics for profit. A physician, though associated with the government may have separate practice in his/her individual private clinic, which is denoted by a visible display board. Such a physician also qualifies as a PP. Only those physicians who were consulted by adult outpatients and were practicing in a clinic with not more than two practicing practitioners were included in the study.

A list of PPs participating in PPM-TB was obtained from the senior treatment supervisor (STS) and the laboratory technician (LT) of one of the DMCs. PPs linked with the programme were approached along with the programme staff to seek appointments for interviews. PPs not included in the list of the TU were approached through a friend who worked as marketing executive of the corporate hospital functioning in the TU area. His personal contacts with a majority of practitioners helped when seeking appointments with PPs not linked with the TB programme. In either case, those willing to participate in the study were included in the study sample. No distinction was made in selecting the PPs based on their system of medical practice, gender, or age. Interviews were conducted till data saturation was achieved. A total of 21 in-depth interviews were held with PPs. On average each interview lasted between 45 minutes to 1 hour.

The interview guide focused on understanding of PPM-TB policy from the respondents’ perspective and how they experienced its implementation at subdistrict level. In addition to in-depth interviews, observations were conducted at field sites. Field notes were maintained to bring out the ‘rich context’ of the data collected.^65^

All interviews were conducted in English. All PPs showed their willingness to record their interviews, except one, wherein detailed notes of the interviews were taken. All interviews were preceded by informed verbal/written consent and all recorded interviews were transcribed word to word using NVivo07 software (QSR International Pty Ltd, Australia). Data coding was done using thematic analysis^66^ and then constantly compared^67^ with data from participant observation and notes from field diaries. Themes were identified using a manual method by back and forth reading of data, without using any computer program. It looked for commonalties, meanings and patterns within the interview transcripts and field notes. These commonalities were then coded, paragraph by paragraph.

This study draws on ethnographic fieldwork, and provides an in-depth exploration of PPM for TB control at the selected field site. This comprehensive understanding in-situ, based on empirical findings, cannot be generalized to the entire Indian setting. However, the findings demonstrate the possible processes and factors affecting relationships at the local level, and further provide deeper insights in explaining those factors.

## Results

The result section has been divided into three broad sections: Profile of PPs, PPs perceptions about their role in PPM-TB, and PPs perceptions towards DOTS.

### Profile of Private Practitioners

A total of 21 PPs were interviewed (Table). Of the interviewed PPs, 13 were qualified allopaths (trained in Western medicine), two of whom were chest specialists. Four of the remaining eight PPs had a degree in Ayurvedic medicine and three were trained in the Unani medical system. Since the Unani system has declined over time, all three were only practicing allopathic medicines, whereas those trained in Ayurveda were engaged in ‘mixed practice.’ The term ‘mixed practice’ here denotes Ayurvedic physicians engaged in prescribing allopathic medicines. Only five out of 13 allopathic practitioners reported of being a member of IMA.

**Table:** Profile of PPs

**PP Types**	**Number**	**DOTS Providers**	**Manage TB own**	**Refer patients to DMC**	**Refer Patient to Private Facility**
MBBS	13	6	9	11	4
BAMS	4	4	1	4	2
BUMS	3	3	0	3	1
RMP	1	1	0	1	1
Total	21	14	10	19	8

Abbreviations: MBBS, bachelor of medicine, bachelor of surgery; BAMS, bachelor of Ayurveda medicine and surgery; BUMS, bachelor of Unani medicine and surgery; RMP, registered medical practitioners; PP, private practitioner; TB, tuberculosis; DMC, designated microscopy centre.

PPs were practicing in small individual clinics, usually a small room divided into consulting room and waiting hall, with wooden partition or curtains. However, four of the PPs had bigger hospital set-ups. Many of these PPs were well-established, and had more than 20 years of experience (ranging from 7 to 30 years) of clinical practice. Two of the chest physicians, and two of the allopathic doctors (MBBS MD) were associated with the public sector as well. Their clinics operated only in the evening hours, whereas the remaining PPs practiced in both the morning as well as the evening. On average, PPs saw a minimum of 20 clients per day (ranging from 10 patients to 100 patients). So far as TB cases were concerned, they not only depended on the client load but also on the location of practice – slum or non-slum. To give an example: An Ayurvedic practitioner has been practicing in a slum area for the last 20 years. On average he saw 40 general patients per day and, he saw ‘monthly two fresh TB patients’ [New cases]. In contrast, a senior allopathic practitioner who had practiced in a non-slum locality for 31 years, has a turnover of 200 cases per day, 5% of whom were suspected of having TB. In all, 19 PPs reported referring their TB suspects to the DMCs: 12 were currently acting as DOTS providers; and two PPs reported that they had stopped being DOTS providers.

### Private Practitioners Perceptions About Their Role in Public-Private Mix-Tuberculosis

The PPs considered themselves to be more accessible and approachable to patients when compared to the government institutions. They perceived themselves to be important healthcare provider, and viewed themselves in different roles within the PPM-TB policy: *‘*to guide patients in taking proper treatment’; ‘to promote their health’; ‘to save patients from disease specific and financial burdens’; ‘to show them the right direction.’

“...*our ultimate goal is achieving good health for the patient…it makes me feel happy if a patient comes and says, “sir I got good results,” that is a good recommendation, a big remuneration, more than that what we can expect...*” (Interview, Ayurvedic Practitioner).

Despite their perceived potential in managing TB patients, however, they felt neglected and undervalued in the implementation of PPM-TB policy, especially in terms of having flexibility to prescribe medicine, and providing supportive treatment to TB patients.

### “We Need Freedom to Prescribe…”

PPs expressed their opinions of the ‘little flexibility’ that RNTCP rendered to them in terms of their role in TB management, that not only decentralises the programme but also de-specialise the skills of PPs.

“*One cannot judge a clinician’s capacity to diagnose, he may not be taking an x-ray to rule out TB, taking an x-ray to rule out pneumonia, to rule out bronchitis, so in fact what happens is RNTCP or any public health programme is, it tries to decentralize and despecialise, so this was an act of despecialisation...”* (Interview, Allopathic Practitioner).

For instance, an allopathic practitioner, who has been practicing in the study area for a long time. The TU being nearer to his clinic, he referred most suspected TB cases to this centre. On most occasions, based on the x-ray results and his professional judgment, he was convinced that the cases he was referring were already TB positive and expected those cases to be put on treatment. However, on many occasions his professional judgments were overruled by the TB staff working at the centre. This perspective from the TB staff made him feel undervalued and, as a result, he stopped sending his cases to the centre.

“…*now when I send a case of two weeks cough, that means, I have already given this patient some treatment for two weeks and since it has not subsided I have referred him. Now what happens, when he goes there they send him back to us, saying that, ‘continue with this medicine for another two weeks.’ So that really puts me off and so I have stopped referring my cases to this DMC. So I think that is how we miss the patients”* (Interview, Allopathic Practitioner).

In India, TB drugs are available across the counter without a prescription slip. Anyone can walk into a private pharmacy to buy TB drugs. PPs expressed their opinion that TB drugs should be made available in all pharmacies and that PPs and patients should have easy access to them. A senior physician who has long-term experience of managing TB patients in his own clinics within the private regimen asserted:

“…*As a private doctor, I am not interested in 250/- Rs. What I need is the freedom to prescribe for my patients. If it is a bad prescription, then definitely the government should intervene. If the prescription is in line with the WHO guidelines the quality of the drugs has to be ensured by the system within the government, which is the drug controller. That is not the concern of somebody else or other agency or WHO…”* (Interview, Allopathic Practitioner).

The policy document states that any patient who has had a cough for two weeks needs to be immediately referred to the DMC to rule out the possibility of TB. PPs, however, found it hard on many occasions to immediately refer patients to the programme. A practitioner mentioned that the *‘very first day I can’t ask any patient to go to the TB centre.’* PPs laid emphasis on the importance of provider-patient relationships stating that, a PP cannot ask any patient to go to the TB centre on the very first day, else they would become panic and lose their trust in PPs.

*“It is quite different here, without asking me these area people they don’t go to any other doctor…accidentally if they consult any other doctor the very next day they get their prescriptions and medicines to me and they continue it only after confirming with me…”* (Interview, Allopathic Practitioner).

If there is a concern for allopathic practitioners about having the freedom to prescribe and manage their patients, then what is it like for the AYUSH practitioners?

### “Our Role Should Go Beyond DOTS Provision…”

In contrast to the MBBS doctors, those practicing AYUSH medicine welcomed the policy of PPM-TB for DOTS and showed willingness to be associated with the programme. However, they emphasised that their role should go beyond DOTS provision. They highlighted the gaps in the programme that have led to a failure to attract practitioners from AYUSH. They saw a weakness in the TB programme that neglects the side effects of treatment and has no proper management of side effects. From the first instance, PPs were aware of the side effects due to the intake of DOTS. They considered DOTS doses to be high, leading to severe side effects which most of the patients were unable to bear and compelled them to discontinue their treatment. A senior Ayurvedic practitioner mentioned a case who ultimately switched from the public to the private sector due to the side effects of DOTS.

“…*after starting the DOTS treatment…the patient had severe gastro intestinal disturbances…so I referred the case back to the DMC…all of a sudden at mid-night the patient developed severe breathlessness, heart burn and vomiting…so the family shifted the patient to the private hospital…the patient consulted a MD physician in the same hospital…I think he started some other anti-TB treatment…”* (Interview, Ayurvedic Practitioner).

AYUSH PPs found that irrespective of the side effects, the programme had limited capacity to deal with them. In contrast, PPs, mostly participating as DOTS providers were efficient in giving side effect treatment to their clients. A senior AYUSH practitioner, who has been associated with the programme for many years, shared his experience of prescribing side treatment to his patients. He mentioned prescribing Liv-52 to all his patients on DOTS regimen as well as those patients taking his own private TB treatment. Liv-52 is an Ayurveda medicine to strengthen the liver and improve its function.

“…*already TB drugs act on the liver. If the liver is healthy then everything is healthy…that’s why we are giving every patient Liv-52 DS…and the results are good. It is very cheap, 30/- Rs per month. So if government people provide Liv-52 or any B complex and protein it will help the patient because he is already anaemic…”* (Interview, Ayurvedic Practitioner).

This practitioner also compared those patients on DOTS in the public sector with those taking DOTS at his clinic. He asserted that those taking DOTS at his clinic along with supportive treatment were much healthier than those taking DOTS without supportive treatment. PPs felt that, irrespective of the important role they can play in managing TB side effects, the public sector’s approach of ‘this is the DOTS box, keep it in your clinic,’ not only undermines their expertise but also prevents them from increasing their involvement in the programme.

### “What Happened? You Are not Sending Cases…”

Mostly MBBS and AYUSH PPs were not very influenced by the financial incentives, and incentives were perceived to be secondary as compared to the benefit their patient would receive from the TB programme in seeking good health. In comparison with the Allopathic and AYUSH practitioners, motivational factor was quite different for ‘unqualified practitioners.’ They considered their association with the programme to be valuable as they got more patients and their relationships with them developed long-term, making these patients their regular clients after completion of their TB treatment. Although they said that financial incentives were secondary for them, field observations showed that financial incentives was the crucial factor in motivating unqualified practitioners to be involved when compared to those individuals with qualifications.

*“I accompanied the STS to the field. In addition to the regular follow up of clients with the PPs, he had an additional task of handing over the honorarium cheques to the PPs. When we entered a g practitioner’s clinic, he looked a bit tired. There was space to sit and due to insufficient light in his clinic everything appeared dull and dark. He asked the STS ‘what happened? Why are you not sending cases?’ I was a bit confused, why was he asking such a question when he himself is supposed to refer the cases to the DMC and not vice-versa? However, later I realised that he meant DOTS patients. For every DOTS patient he was getting Rs. 250 ($US4). The STS right then handed him a cheque for Rs. 1750 ($US28.3) for the seven cases for whom he had provided DOTS in the last year”* (Excerpt from Field Diary, Thursday, May 26, 2011).

This also shows that unqualified PPs were willing to work as DOTS providers and encouraged staff to send more DOTS patients, considering the financial benefits behind it.

#### Private Practitioners Perceptions Towards DOTS

In the previous section, we came across factors that dissuaded the PPs from getting fully involved in implementation of PPM-TB. In this section, we will highlight that in addition to those factors, the PPs had contrasting perceptions about the different components of TB programme that demonstrated public sectors dominance in overall implementation of DOTS strategy.

#### “Tuberculosis Programme Is Public Sector Driven…”

The entire process of involving a PP in the TB programme under the PPM-TB policy was considered to be ‘public sector-driven’ by most of the practitioners; the diagnosis was with the public sector and the medicine was with the public sector. PPs were viewed simply as referral points to gain cases. Irrespective of the contribution PPs made, the credit was always considered to be taken by the government.

“…‘*If you give cases to PPs, our credit will disappear’…means RNTCP doesn’t want to give their cases to PPs for management because then, how will they take the credit for the programme?”* (Interview, IMA Representative).

PPs assertion that *‘TB programme is public sector driven…’* was overall based on the following components.

#### i. Intermittent Verses Daily DOTS

The RNTCP recommends standardised alternate day DOTS to all patients. However, the private sector never favoured the standardised alternate day DOTS regimens over case-specific daily doses. The RNTCP has stood out as going against the traditional pattern of medicine prescription in India and TB is the ‘only communicable disease with intermittent therapy.’ A senior chest physician who is associated with the public sector, but also has his own private practice, expressed his opinion as follows:

“…*See, if you are a diseased man treatment will be a continuous. Take a tablet three times daily that is by convention and tradition. It is there, it is the belief, and both the providers and patients are used to that belief, so when you try to engage the private sector, private behaviour of prescribing and patient behaviour of taking medicines need to be considered…”* (Interview, Senior Chest Physician).

In addition to ‘patient behaviour of taking medicines,’ another reason that was flagged by most of the PPs was the ‘pill load.’ PPs asserted that daily DOTS, as compared to alternate DOTS would cause less harm to the patients.

“*See this formation of DOTS schedule is absolutely wrong, one day you give high dose that is the peak level of drug, means you give double dose but the next day you give nothing. In daily regimen you give balanced drug every day that means you reduce the scope for resistance of the bacteria…it’s not very high one day and nothing the other…”* (Interview, Allopathic Practitioner).

#### ii. “Observation” of DOTS Is Difficult

The WHO initiated DOTS policy recommends that the swallowing of all doses of TB drugs by the patient should be supervised either by a frontline health worker or by a community or family member, work colleague or private provider nominated by the patient and the health provider. PPs felt that the RNTCP claimed to have a strong monitoring component in place and that PPs were often criticised by programme and staff members for not having a proper monitoring system in place to observe their own TB patient treatment. PPs, however, questioned the feasibility of DOTS. Even those PPs who had DOTS box in their clinics failed to observe their DOTS patients swallowing the TB medicine in front of them. A senior doctor who was associated with the public sector and also had his own private clinic running in the late evenings for almost 25 years, referred his cases regularly to the nearest DMC and mentioned that he had good cooperation from the staff at the DMC located near his clinic. However, he said that observing DOTS was only a constraint.

“…*The only constraint is observing the DOTS…a good number of people [patients] are sitting in a small room, so it is very difficult to observe the DOTS…That is the only constraint that I have faced in my clinic…”* (Interview, Allopathic Practitioner).

Field observations at various DOTS centres revealed that all private clinics had an attendant, usually a non-medical person who helped the practitioner in the day to day functioning of the clinic. The PP rarely came into contact with the patient who visited the clinic to take his DOTS strip of medication. The patient directly got in touch with the attendant who handed over the strips, rarely bringing the observation component into practice. Furthermore, observations at the DMCs were not different from those occurring at the private clinics. Due to a high patient turnover, the health staff at the DMC failed to observe the patients swallowing their medicines, only the first one or two doses were observed; whereas the remaining occasions the drugs were simply handed over to the client.

*“…Multi-purpose health supervisor (MPHS) was on leave today, so I thought of helping the LT in distributing the DOTS strip. Later in the afternoon, a lady came and asked for her DOTS strip for phase one. I pulled out a strip and handed it over to her, she replied, ‘it’s only one strip, I need two more.’ I said, you need to swallow this and come back the day after tomorrow. She was a bit upset over this and said to me, ‘Are you a new sir? The other sir [MPHS] always gives me three strips.’ I looked at the LT and he just nodded his head; that meant, I should give her two more strips…”* [Excerpt from Field Diary, Tuesday, May 31, 2011].

#### iii. No Chest Physicians at Tuberculosis Centres

A further gap identified by physicians was the program’s undue attention to the disease rather than to the patient as a whole. They considered that a TB patient needs attention from a holistic perspective and each case needed to be understood in relation to his body function. PPs linked TB management and chest physicians (CPs) together, and shared their experience of referring their clients to private CPs. A very senior Ayurvedic practitioner highlighted the importance of having proper CPs in the programme who can manage the TB cases holistically.

“…*A chest physician looks after a TB patient from all angles. He gives TB drugs but also monitors and caters to his side effects simultaneously. This is the loop hole in the RNTCP…”* (Interview, Ayurvedic Practitioner).

PPs observed that most of the CPs who had private clinics were either full-time professors at government teaching colleges/hospitals or retired professors. PPs felt that such CPs who had long-term experience of dealing with chest diseases needed to be hired by the programme, or the programme should request them to volunteer their services. PPs felt that in the absence of CPs, there was an over reliance on the frontline staff who themselves decided who to start on treatment. In the absence of proper CPs, and to avoid the conflicts with TB staff, PPs had developed a link with nearby private CPs for their private patients.

“*See, our aim is to give better treatment to the patient...because in DMCs they don’t have chest physicians...So in that case whenever you need a chest physician, it’s better to advise a patient to see a chest physician…sometimes I advise them to go to a private chest physician…”* (Interview, Ayurvedic Practitioner).

Interestingly, interactions with three CPs (who were also government employees) revealed that in their private clinics they were the least supportive of the DOTS strategy and were more in favour of prescribing their own treatment to the clients.

“...*See the pill load in DOTS; there are seven tablets. The patient has to take it thrice a week. Daily treatment will reduce the pill load; taking few tablets at one time will reduce the side effects...”* (Interview, Senior Chest Physician).

#### iv. More Concentration on Pulmonary Tuberculosis

PPs considered it as ‘a loop hole in the programme,’ wherein the programme failed to treat extra pulmonary, TB cases. PPs felt that, on the one hand the programme advocates for referral from them, but on the other hand when referred, and if a case turns out to be extra pulmonary, then it is given less attention.

“…*Depending on sputum examination…I don’t think, because there are many cases of extra pulmonary tuberculosis [EP-TB] also. Now they don’t care for EP-TB…for EP also they will ask for sputum examinations If lymphadenitis [Inflammation of a lymph node] is there or if patient is with Cox Spine [Spine TB], they don’t give. Government people, they don’t give [treatment], unless and until they are told from chest hospitals…”* (Interview, Allopathic Practitioner).

Such instances made PPs strongly assert that the TB programme was highly government-driven, making PPs feel isolated, not only at the field level but also at the level of policy decisions and planning. As a result, although PPs felt responsible towards TB patients by participating in PPM-DOTS, they found it difficult to perceive themselves as a ‘partner to the programme.’

## Discussion

This study explored the national PPM policy for TB control in India by focusing on the perceptions and experiences of the PPs engaged in implementing DOTS programme at the subdistrict level. The main focus was not on assessing policy outcomes in terms of successes or failures, but to understand the concerns of PPs, the challenges they face, and the implication of these findings on their continued involvement with the programme. A strength of this study lies in the insights it provides through in-depth interactions with PPs, and also through the daily observations made in the field. The study does not look into the day to day management of TB patients by the PPs in their clinics.

The results highlight the constraints that individual PP’s face which affect their relationships with the programme and with other practitioners. Although DOTS was agreed to in principle, in practice PPs held a negative attitude towards the policy. Similar responses from private stakeholders have been documented by Atun et al^68^ in their study on DOTS implementation in Russia.The idea of referring those suspected of having TB to government health facilities, has challenged the traditional medical role of PPs, giving them less freedom to diagnose or to decide on appropriate referrals.

In the RNTCP, an algorithm is followed for the diagnosis of TB cases.^69^ If the suspect shows sputum negative results during the first screening, then they receive symptomatic treatment and broad spectrum antibiotics for 10-14 days. If the symptoms persist for 15 days, a repeat sputum investigation is performed and, if necessary, a chest x-ray. Medical practitioners have the authority to make treatment decisions based on their individual professional judgement and expertise. While evaluating Starr’s^70^ analysis of the impact on medicine of healthcare, Halpern stated, ‘Starr maintains that medicine’s professional standing is rooted in its cultural authority, a form of legitimacy that enables physicians to make judgments of meaning and value and have these held to be true and valid.’^71^ The traditional Indian clinical approach to TB diagnosis under the NTP has been through the use of x-rays.^24^ In contrast to this, the RNTCPs WHO recommended DOTS strategy advocates to use sputum microscopy for TB diagnosis and for some PPs this has taken away their autonomy. The sputum smear microscopy is simple, rapid, inexpensive,^72^ and more ‘specific’ when compared to chest x-ray.^73,74^ However, the wide spread use of x-ray by PPs, and their ability to make clinical judgments cannot be ignored if their participation needs to be sustained. In recent years, development has occurred around rapid diagnosis for TB using techniques such as the Gene Xpert MTB/RIF. Although its availability among private labs is increasing,^75^ there is still a dearth of information around its use and preference among PPs.

In contrast to a daily regimen, the DOTS strategy advocates standardised alternate day TB drugs to all patients. In the field of PPM-TB, where PPs are invited to be a partner, the RNTCP has constantly advocated for this new DOTS strategy. This has led to extensive debate, and the private sector has been consistent in criticising standardised alternate treatment regimes, as compared to case-specific tailored daily doses.^76^ As mentioned by one of the CP in this study: *‘…private behaviour of prescribing and patient behaviour of taking medicines need to be considered….’* This belief might be considered to be ‘common sense’ or even an ultimate truth for most medical practitioners and individuals in the society. It also emphasises the need for considering the health beliefs and social norms of the community if the health systems is going to be efficient.^77^ The policy’s lack of flexibility in allowing PPs to prescribe TB treatment on their own, or using their own system of medicine as a supportive treatment (in the case of AYUSH PPs), can be seen as a process of undervaluing their educational capability as well as their professional skills. Similar experiences of PPs feeling undervalued and demotivated in the TB programme have been reported in other studies conducted in India and elsewhere.^68,78^

None of the public sector associated CPs supported ‘observation’ of DOTS in their private practices. PPs asserted that the policy-makers and national programme managers have ignored the challenges faced by implementers in ‘observing’ DOTS, and have continued to push the component of ‘observation.’ A Cochrane review has shown that patients observed while taking their TB drugs did not improve the cure rate compared with patients without direct observation of treatment.^79^ The hard and fast rule or ‘dogma’ of ‘observation’ also reflects the programme’s mistrust of patients, and makes the whole aspect of ‘observation’ more technical, thus, missing out on the importance of the human aspects of relationship.

In addition, the priority within DOTS is given to the most infectious cases, ie, the smear positive cases and often, the smear negative cases are taken less seriously. This means that extra pulmonary TB cases get little attention and are often neglected.^80^

There was a serious resistance from PPs over the lack of CPs within the TB programme. Interestingly, in the absence of CPs at the TB centres, PPs had developed a link for their patients with nearby private CPs. A recent study conducted in Maharashtra has shown that most of the private CPs were generally complying with the current guidelines for management of drug resistance TB.^81^ This provides a further opportunity for TB programmes to involve CPs in the diagnosis and treatment of TB patients, as well as an opportunity to strengthen the private-private relationships.

An overemphasis on sputum, combined with the strong recommendation for the alternate day regimen for treatment has not only side-lined the use of x-ray and daily regimens, but in a deeper sense has interfered with the PPs’ perceived freedom and professional autonomy to diagnose and treat their TB patients. It appears not only to hinder the reach of the programme, but also makes it difficult for practitioners to participate and to contribute.

These challenges and constrains experienced by PPs also reflects the ‘inherent contradiction in the public health approach to the control of infectious diseases like TB.’^82^ In public health, populations gets prioritised over individuals.^83^ Both the approaches are essential, and there needs to be a balance. One way of achieving it could be through the wider dissemination of International Standards for TB Care (ISTC) among PPs, and through allowing the PPs more freedom to prescribe different regimens that would be confined within the wider guidelines of the ISTC.^84^ The RNTCP–IMA led training programmes for allopathic practitioners have adopted the ISTC,^85^ however, it was surprising to note that not all allopathic practitioners were members of the IMA. These factors need to be considered when planning future dissemination/training programmes for practitioners.

The PPM-TB policy has categorised all types of PPs under one broad group as ‘private providers.’ This single label has not only overlooked the hierarchy amongst practitioners,^86^ but has also ignored the different perspectives and potential of each system of medicine and the contribution they can bring to TB control efforts. The PPs are often perceived as ‘money-minded’ or ‘with a business-minded approach,’ and financial incentives are considered one of the best ways to involve them in any health programme.^5^ The current PPM-TB policy document tends to see all practitioners through the same lens of financial incentives. In this study, however, the interactions with PPs revealed that their motivation was not purely incentive-based; rather there was a variation across types of providers in terms of their need for incentives. MBBS PPs were not very influenced by the financial incentives, and financial incentives were not the best means to enhance their involvement. Likewise, for those following the AYUSH system of medicine, financial incentives were secondary as compared to the benefit their patients would receive from the TB programme in seeking good health. Similar experiences are shared by Khan et al^86^ based on their experience and work with PPM initiatives in South Asia.

One PPs assertion that, *‘TB programme is public sector driven…’* also reflects the overpowering authority of the public sector over the private sector. In their study of an NGO-Government partnership in India, Baru and Nundy^87^ reported that the public sector is the major player and defines the terms and conditions, whereas the private sector plays the minor role, and has to consent or negotiate terms and conditions. The DOTS strategy (alternate day drugs) was derived from the international TB policy and has been incorporated into the RNTCP without taking into consideration the local context – which in our study area demonstrated a large private medical sector with PPs that practices case-specific treatment and the use of tailored daily doses of medication. As a result, although the WHO’s stop TB strategy has been instrumental in bringing the PPM-TB partnership policy into the field at the local, it has not made a significant impact in building trust and relationships across the different sectors.

Scholars investigating the policy transfer of DOTS have commented that the ‘top-down internationally driven policy changes may lead to apparent policy transfer, but not necessarily to successfully implemented programmes.’^88^ If the PPM-TB policy initiatives continue to remain top-down and become too technical in their implementation, then the policy will continue to widen the barriers of mistrust within the TB programme.^89^ This will also pose future threats to other partnership initiatives across the public and private sectors. If national policies are to be effective at the subdistrict level, then the TB programme needs to be flexible enough. The experiences of people implementing DOTS and their abilities to deal with the patients need to be recognised accordingly, and supported with appropriate strategies for ensuring the success of TB control efforts.

Nonetheless there is a hope for achieving sustainable partnerships with PPs, as the country moves towards achieving the objectives laid out in the WHO’s ‘post-2015 TB strategy.’^18,22^ In 2012, the Indian Government adopted a policy on ‘mandatory notification of TB cases’ that gives PPs the flexibility to have their TB patients tested at the DMC, but to manage treatment on their own, provided they register their TB cases.^90^ This policy is still in its preliminary stages and there is currently no evidence on how relationships between providers and the programme can be enhanced to strengthen the health systems. Additionally, since November 2013 the Department of AYUSH has been elevated as an independent ministry. One of its key functions is to generate awareness about the efficacy of the AYUSH system domestically and internationally. Although there are initiatives within the TB programme to involve AYUSH practitioners, their potential needs to be further explored and expounded.

## Conclusion

Strong health systems are a prerequisite to improve health outcomes and to accelerate progress towards achieving the national target of elimination of TB by 2020. Any strategy employed towards strengthening health systems in India, however evidenced based, may fail if the concerns and needs of the PPs continue to be ignored within the policy framework. Understanding the professional level of individual practitioners is crucial and needs to be supported with an appropriate incentive structures in order for their long-term involvement in the programme to be ensured. The untapped potential of different medical systems needs to be recognised, trusted and supported with the creation of suitable policy measures in the ‘post-2015 TB strategy.’

## Acknowledgements

This paper idea developed during the workshop on ‘writing policy brief’ organised as a part of Emerging Voices for Global health (EV4GH - 2014) training programme in Cape Town, South Africa. We are grateful to all participants of this study, for their precious time and valuable insights. Without such privileged access, this research would never have been possible. We wish to acknowledge participants by name, but out of ethical considerations, will refrain from doing so. Thanks to Dr. Abhay Kudale (MAAS-CHRD) for his constructive comments on earlier drafts of this manuscript. Sincere thanks to the Commonwealth Scholarship Commission (CSC), London, UK for providing the financial support that made this research possible.

## Ethical issues

Ethical clearance for this study was obtained from the Institutional Ethics Committee of the London School of Hygiene and Tropical Medicine (LSHTM), London, UK and the Local Ethics Committee, India.

## Competing interests

Authors declare that they have no competing interests.

## Authors’ contributions

SS made substantial contributions to the conception and design of the paper. He initiated first draft of this manuscript, coordinated with other coauthors worked on their comments/suggestions and led subsequent drafts up to this finalized manuscript. KS gave inputs, edited drafts and contributed to critical revision of the manuscript for important intellectual content. JDP helped to develop the first draft of the manuscript and contributed through critical revisions to the final draft.

## Authors’ affiliations

^1^Department of Global Health and Development, London School of Hygiene and Tropical Medicine, London, UK. ^2^The Maharashtra Association of Anthropological Sciences, Centre for Health Research and Development (MAAS-CHRD), Savitribai Phule Pune University, Pune, India. ^3^Public Health Foundation of India, New Delhi, India. ^4^Departments of Clinical Research and Global Health and Development, London School of Hygiene and Tropical Medicine, London, UK.

## Key messages

**Implications for policy makers**

- Public-private mix-tuberculosis (PPM-TB) policy has categorised all types of private practitioners (PPs) (irrespective of system of practice) under one broad group as ‘private providers.’ The policy document tends to see all practitioners through the same lens of financial incentives. The interactions with PPs, however, revealed that their motivation was not purely incentive-based; rather there was a variation across types of providers in terms of their need for incentives.
- Understanding the professional level of individual practitioners is crucial, and needs to be supported with an appropriate incentive structures in order for their long-term involvement in the programme to be ensured.
- PPM-TB policy has ignored the potential of alternative systems of medicine and the contribution they can bring to TB control efforts.
- PPM-TB policy’s lack of flexibility in allowing PPs to prescribe TB treatment on their own, or using their own system of medicine as a supportive treatment, affects PP involvement.
- The untapped potential of different medical systems needs to be recognised, trusted and supported with suitable policy measures in the ‘post- 2015 TB strategy.’

**Implications for public**

Public and private health sectors both play a crucial role in the health systems of low- and middle-income countries (LMICs), and need continuous efforts to build effective partnerships for strengthening health systems. The Ministry of Health and Family Welfare (MoHFW) in India encourages PPPs, by developing national policies and guidelines for the engagement of all healthcare providers in different National Health Programmes. However, achieving a sustained partnership between the public and private health sectors, through sustaining interest, motivation and involvement of private practitioners (PPs) in disease programmes (both communicable and non-communicable), continues to be a major challenge for the concerned health authorities. This paper draws on the constraints and challenges faced by PPs in the implementation of the public-private mixtuberculosis (PPM-TB) policy in India.

## References

[1] Berendes S, Heywood P, Oliver S, Garner P (2011). Quality of private and public ambulatory health care in low and middle income countries: systematic review of comparative studies. PLoS Med.

[2] Konde-Lule J, Gitta SN, Lindfors A, Okuonzi S, Onama VO, Forsberg BC (2010). Private and public health care in rural areas of Uganda. BMC Int Health Hum Rights.

[3] Ha NT, Berman P, Larsen U (2002). Household utilization and expenditure on private and public health services in Vietnam. Health Policy Plan.

[4] Wang Y. Public-Private Partnerships in the Social Sector: Issues and Country Experiences in Asia and the Pacific. Vol ADBI Policy Paper No. 1. Tokyo: Asian Development Bank Institute; 2000.

[5] Raman AV, Björkman JW. Public-Private Partnerships in Health Care in India: Lessons for developing countries. Taylor & Francis; 2008.

[6] Bennett S, Hygiene LS. The Mystique of Markets: Public and Private Health Care in Developing Countries. London: Department of Public Health & Policy, London School of Hygiene & Tropical Medicine; 1991.

[7] Brugha R, Zwi A (1998). Improving the quality of private sector delivery of public health services: challenges and strategies. Health Policy Plan.

[8] Uplekar MW (2000). Private health care. Soc Sci Med.

[9] Mills A, Brugha R, Hanson K, McPake B (2002). What can be done about the private health sector in low-income countries?. World Hosp Health Serv.

[10] Berman P, Laura R (1996). The role of private providers in maternal and child health and family planning services in developing countries. Health Policy Plan.

[11] Berman P (2001). Getting more from private health care in poor countries: a missed opportunity. Int J Qual Health Care.

[12] World Health Organization (WHO). WHO Assembly Resolution: Strengthening the capacity of governments to constructively engage the private sector in providing essential health-care services. 63rd World Health Assembly, A63/25. Geneva: World Health Organization; 2010.

[13] International Institute For Population Sciences (IIPS). National Family Health Survey (NFHS-3), 2005–06. India Mumbai: IIPS and Macro International; 2007.

[14] Central Bureau of Health Intelligence (CBHI). Human Resource in Health Sector - National Health Profile (NHP) of India. http://www.cbhidghs.nic.in/index2.asp?slid=1256&sublinkid=1163. Accessed February 19, 2014. Published 2012.

[15] Reddy KS, Patel V, Jha P, Paul VK, Kumar AK, Dandona L (2011). Towards achievement of universal health care in India by 2020: a call to action. Lancet.

[16] Sachdeva KS, Kumar A, Dewan P, Kumar A, Satyanarayana S (2012). New vision for Revised National Tuberculosis Control Programme (RNTCP): universal access - “reaching the un-reached. ” Indian J Med Res.

[17] Ministry of Health and Family Welfare (MoHFW). Universal access to TB Care- A practical guide for programme managers. New Delhi: Central Tuberculosis Division, Directorate General of Health Services; 2010.

[18] Uplekar M. Place of PPM in the post-2015 TB strategy. http://www.who.int/tb/careproviders/ppm/PPM_post_2015_Strategy.pdf. Published 2013.

[19] Revankar CR (2008). Public-private partnership in public health programmes in India. Health Administrator.

[20] Wells WA, Uplekar M, Pai M (2015). Achieving systemic and scalable private sector engagement in tuberculosis care and prevention in Asia. PLoS Med.

[21] World Health Organization (WHO). Global Tuberculosis Report 2013. Geneva: WHO; 2013.

[22] World Health Organization (WHO). Global Tuberculosis Report 2010. Geneva: WHO; 2010.

[23] Ministry of Health and Family Welfare (MoHFW). TB INDIA 2013 RNTCP Status Report. New Delhi: Central TB Division, MoHFW; 2013.

[24] Kumar P (2005). Journey of tuberculosis control movement in India: National Tuberculosis Programme to revised National Tuberculosis Control Programme. Indian Journal of Tuberculosis.

[25] Khatri GR, Frieden TR (2002). Controlling tuberculosis in India. N Engl J Med.

[26] Ministry of Health and Family Welfare (MoHFW). TB INDIA 2011 RNTCP Status Report. New Delhi: Central TB Division, MoHFW; 2011.

[27] World Health Organization (WHO). Involving Private Practitioners in Tuberculosis Control: Issues, interventions, and emerging policy framework. Geneva: WHO; 2001.

[28] Uplekar M, Juvekar S, Morankar S, Rangan S, Nunn P (1998). Tuberculosis patients and practitioners in private clinics in India. Int J Tuberc Lung Dis.

[29] Department For International Development (DFID). Summary Report: Health Care Provider Survey in Andhra Pradesh, India. Impact Assessment for HIV/STI Prevention Programmes: Baseline Report Series. Andhra Pradesh: Department For International Development (DFID), AP State AIDS Control Society (APSACS) and Family Health International (FHI); 2001.

[30] MAAS. Can the Private and Public Sectors Collaborate for Effective Management of TB, HIV and Co-infection? A Situational Analysis in Hyderabad City, Andhra Pradesh. http://www.lshtm.ac.uk/dfid/targets/HIV-TB-PPM-Situation-Analysis-MAAS-CHRD-Dissemination%20Flyer.pdf. Accessed January 20, 2008. Published 2007.

[31] Uplekar MW, Rangan S (1993). Private doctors and tuberculosis control in India. Tuber Lung Dis.

[32] Uplekar MW, Shepard DS (1991). Treatment of tuberculosis by private general practitioners in India. Tubercle.

[33] Prasad R, Nautiyal RG, Mukherji PK, Jain A, Singh K, Ahuja RC (2002). Treatment of new pulmonary tuberculosis patients: what do allopathic doctors do in India?. Int J Tuberc Lung Dis.

[34] Singla N, Sharma PP, Singla R, Jain RC (1998). Survey of knowledge, attitudes and practices for tuberculosis among general practitioners in Delhi, India. Int J Tuberc Lung Dis.

[35] Kelkar-Khambete A, Kielmann K, Pawar S (2008). India’s Revised National Tuberculosis Control Programme: looking beyond detection and cure. Int J Tuberc Lung Dis.

[36] Atre SR, Mistry NF (2005). Multidrug-resistant tuberculosis (MDR-TB) in India: an attempt to link biosocial determinants. J Public Health Policy.

[37] Udwadia ZF, Pinto LM, Uplekar MW (2010). Tuberculosis management by private practitioners in Mumbai, India: has anything changed in two decades?. PLoS One.

[38] Sheikh K, Porter J, Kielmann K, Rangan S (2006). Public-private partnerships for equity of access to care for tuberculosis and HIV/AIDS: lessons from Pune, India. Trans R Soc Trop Med Hyg.

[39] Bhat R (1996). Regulation of the private health sector in India. Int J Health Plan Manage.

[40] Fochsen G, Deshpande K, Diwan V, Mishra A, Diwan VK, Thorson A (2006). Health care seeking among individuals with cough and tuberculosis: a population-based study from rural India. Int J Tuberc Lung Dis.

[41] World Health Organisation (WHO). Informal consultation on private practitioners involvement in control of communicable diseases with a focus on tuberculosis. Geneva: WHO; 2000.

[42] World Health Organisation (WHO). Public-private mix for DOTS: report of the second meeting of the PPM subgroup for DOTS expansion. Geneva: WHO; 2004.

[43] Chauhan LS (2007). Public-private mix DOTS in India. Bull World Health Organ.

[44] Ministry of Health and Family Welfare (MoHFW). Involvement of non-governmental organizations in the Revised National Tuberculosis Control Programme. New Delhi: Central TB Division, MoHFW; 2001.

[45] Ministry of Health and Family Welfare (MoHFW). Involvement of Private Practitioners in the Revised National Tuberculosis Control Programme. New Delhi: Central TB Division, MoHFW; 2002.

[46] Agarwal SP, Sehgal S, Lal SS. Public-Private Mix in the Revised National TB Control Programme. In: Agarwal SP, Chauhan LS, eds. Tuberculosis Control in India. India: Directorate General of Health Services, Ministry of Health and Family Welfare; 2005.

[47] Murthy KJ, Frieden TR, Yazdani A, Hreshikesh P (2001). Public-private partnership in tuberculosis control: experience in Hyderabad, India. Int J Tuberc Lung Dis.

[48] Uplekar M, Lönnroth K. Engaging Private Providers in Tuberculosis Control: Public-Private Mix for DOTS. In: Raviglione MC, ed. Reichman and Hershfield’s Tuberculosis: A Comprehensive, International Approach. New York: Taylor & Francis; 2006.

[49] Dewan PK, Lal SS, Lonnroth K (2006). Improving tuberculosis control through public-private collaboration in India: literature review. BMJ.

[50] Engel N, van Lente H (2013). Organisational innovation and control practices: the case of public–private mix in tuberculosis control in India. Soc Health Illn.

[51] Ministry of Health and Family Welfare (MoHFW). TB INDIA 2010 RNTCP Status Report. New Delhi: Central TB Division, MoHFW; 2010.

[52] Ministry of Health and Family Welfare (MoHFW). Revised Schemes for NGOs and Private Providers New Delhi: Central TB Division, MoHFW; 2008.

[53] Sahu S, Chauhan LS. The Role of WHO in the Successful Implementation and Expansion of the DOTS Programme in India. In: Agarwal SP, Chauhan LS, eds. Tuberculosis Control in India. India: Directorate General of Health Services, Ministry of Health and Family Welfare; 2005.

[54] Frieden TR, Khatri GR (2003). Impact of national consultants on successful expansion of effective tuberculosis control in India. Int J Tuberc Lung Dis.

[55] Ministry of Health and Family Welfare (MoHFW). TB INDIA 2009 RNTCP Status Report. New Delhi: Central TB Division, MoHFW; 2009.

[56] Lal SS, Sahu S, Wares F, Lonnroth K, Chauhan LS, Uplekar M (2011). Intensified scale-up of public-private mix: a systems approach to tuberculosis care and control in India. Int J Tuberc Lung Dis.

[57] Ministry of Health and Family Welfare (MoHFW). TB INDIA 2006 RNTCP Status Report. New Delhi: Central TB Division, MoHFW; 2006.

[58] Ministry of Health and Family Welfare (MoHFW). TB INDIA 2007 RNTCP Status Report. New Delhi: Central TB Division, MoHFW; 2007.

[59] Ministry of Health and Family Welfare (MoHFW). TB INDIA 2008 RNTCP Status Report. New Delhi: Central TB Division, MoHFW; 2008.

[60] Ministry of Health and Family Welfare (MoHFW). TB INDIA 2012 RNTCP Status Report. New Delhi: Central TB Division, MoHFW; 2012.

[61] Goenka S (2006). Collaboration or communication with private providers? Conclusions based on insufficient evidence. BMJ.

[62] Mahendradhata Y, Lambert ML, Boelaert M, Van der Stuyft P (2007). Engaging the private sector for tuberculosis control: much advocacy on a meagre evidence base. Trop Med Int Health.

[63] Ministry of Health and Family Welfare (MoHFW). TB INDIA 2014 RNTCP Status Report. New Delhi: Central TB Division, MoHFW; 2014.

[64] Reeves S, Kuper A, Hodges BD (2008). Qualitative research methodologies: ethnography. BMJ.

[65] Bernard HR, Bernard HR. Research Methods in Anthropology: Qualitative and Quantitative Approaches. AltaMira Press; 2006.

[66] Guest G, MacQueen KM, Namey EE. Applied Thematic Analysis. SAGE Publications; 2011.

[67] Charmaz K. Constructing Grounded Theory: A Practical Guide Through Qualitative Analysis. SAGE Publications; 2006.

[68] Atun RA, Baeza J, Drobniewski F, Levicheva V, Coker RJ (2005). Implementing WHO DOTS strategy in the Russian Federation: stakeholder attitudes. Health Policy.

[69] Ministry of Health and Family Welfare (MoHFW). Training Module for Medical Practitioners. New Delhi: Central TB Division, MoHFW; 2010.

[70] Starr P. The Social Transformation of American Medicine: The Rise of a Sovereign Profession and the Making of a Vast Industry. New York: Basic Books; 1982.

[71] Halpern SA (2004). Medical authority and the culture of rights. J Health Polit Policy Law.

[72] Desikan P (2013). Sputum smear microscopy in tuberculosis: Is it still relevant?. Indian J Med Res.

[73] Duanmu HJ, Zheng SH, Xu B, Fu CW (2005). Improved case finding by using sputum examination in pulmonary tuberculosis suspects with clinical symptoms (Chinese). Zhonghua Jie He He Hu Xi Za Zhi.

[74] Bawri S, Ali S, Phukan C, Tayal B, Baruwa P (2008). A study of sputum conversion in new smear positive pulmonary tuberculosis cases at the monthly intervals of 1, 2 & 3 month under directly observed treatment, short course (dots) regimen. Lung India.

[75] Puri L, Oghor C, Denkinger CM, Pai M (2016). Xpert MTB/RIF for tuberculosis testing: access and price in highly privatised health markets. Lancet Glob Health.

[76] Harper I (2009). Harper INational tuberculosis control programmes of Nepal and IndiaAre they using the correct treatment regimens?. J Health Stud.

[77] Barnhoorn F, Adriaanse H (1992). In search of factors responsible for noncompliance among tuberculosis patients in Wardha District, India. Soc Sci Med.

[78] Engel N. Innovation dynamics in Tuberculosis control in India: the shift to new partnerships (Working paper series). The Netherlands: United Nations University - Maastricht Economic and social Research and training centre on Innovation and Technology ; 2009.

[79] Volmink J, Garner P (2007). Directly observed therapy for treating tuberculosis. Cochrane Database Syst Rev.

[80] Prakasha SR, Suresh G, D’sa IP, Shetty SS, Kumar SG (2013). Mapping the Pattern and Trends of Extrapulmonary Tuberculosis. J Glob Infect Dis.

[81] Dholakia Y, Quazi Z, Mistry N (2012). Drug-resistant tuberculosis: study of clinical practices of chest physicians, Maharashtra, India. Lung India.

[82] Hurtig AK, Porter JD, Ogden JA (1999). Tuberculosis control and directly observed therapy from the public health/human rights perspective. Int J Tuberc Lung Dis.

[83] Porter J, Kielmann K (2003). TB Control in India: the need for research in policy and decision making. Journal of the Indian Society of Health Administrators.

[84] Hopewell PC, Fair EL, Uplekar M (2014). Updating the International standards for tuberculosis care Entering the era of molecular diagnostics. Ann Am Thorac Soc.

[85] Achanta S, Jaju J, Kumar AM (2013). Tuberculosis management practices by private practitioners in Andhra Pradesh, India. PLoS One.

[86] Khan MS, Salve S, Porter JD (2015). Engaging for-profit providers in TB control: lessons learnt from initiatives in South Asia. Health Policy Plan.

[87] Baru RV, Nundy M (2008). Blurring of boundaries: public-private partnerships in health services in India. Econ Polit Wkly.

[88] Ogden J, Walt G, Lush L (2003). The politics of ‘branding’ in policy transfer: the case of DOTS for tuberculosis control. Soc Sci Med.

[89] De Costa A, Johansson E, Diwan VK (2008). Barriers of mistrust: public and private health sectors’ perceptions of each other in Madhya Pradesh, India. Qual Health Res.

[90] Ministry of Health and Family Welfare (MoHFW). Notification of TB Cases, Z-28015/2/2012-TB. New Delhi: MoHFW; 2012.

